# Plaque Prolapse in Carotid Artery Stenting: Mechanisms, Imaging and Device-Based Prevention

**DOI:** 10.3390/medicina62071235

**Published:** 2026-06-26

**Authors:** Luca Galassi, Leonardo Pasquetti, Federica Facchinetti, Rebecca Magugliani, Elena Goldoni, Edoardo Pasqui, Giovanni Nano, Gianmarco de Donato

**Affiliations:** 1 Postgraduate School of Vascular and Endovascular Surgery, University of Milan, Via Festa del Perdono 7, 20122 Milan, Italy; 2 Vascular Surgery, Department of Medicine, Surgery and Neuroscience, University of Siena, Viale Bracci, 53100 Siena, Italy; l.pasquetti@student.unisi.it (L.P.); edoardo.pasqui1@unisi.it (E.P.); dedonato@unisi.it (G.d.D.); 3 School of Medicine and Surgery, University of Milan-Bicocca, Via GB Pergolesi 39, 20900 Monza, Italy; f.facchinetti4@campus.unimib.it; 4 Cardiotoracovascular Research Unit, IRCCS Galeazzi Sant’Ambrogio Hospital, Via Cristina Belgioioso 57, 20157 Milan, Italy; rebecca.magugliani@grupposandonato.it; 5 School of Medicine and Surgery, University of Milan, Via Festa del Perdono 7, 20122 Milan, Italy; elena.goldoni@studenti.unimi.it; 6 Vascular Surgery Unit, IRCCS Policlinico San Donato, San Donato Milanese, 20097 Milan, Italy; giovanni.nano@unimi.it; 7 Department of Biomedical Sciences for Health, University of Milan, Via Festa del Perdono 7, 20122 Milan, Italy

**Keywords:** carotid artery stenting, plaque prolapse, vulnerable plaque, dual-layer micromesh stent, optical coherence tomography, cerebral embolization, CGuard, Roadsaver

## Abstract

Carotid artery stenting (CAS) is an established revascularization option for patients with carotid disease at high surgical risk. Periprocedural cerebral embolization remains a clinical concern, and plaque prolapse, the extrusion of atherosclerotic material through the stent struts into the lumen, has emerged as an actionable mechanism directly linked to embolic events and adverse neurological outcomes. This narrative review provides a structured, practice-oriented framework addressing one specific question: how can plaque prolapse be prevented during CAS? Drawing on prospective registries, comparative cohort studies and intravascular imaging analyses based on optical coherence tomography (OCT) and intravascular ultrasound (IVUS), we discuss the main determinants of prolapse risk, plaque morphology, procedural variables and device selection, converging on dual-layer micromesh stent (DLMS) technology as the most advanced device-based solution available. Pooled clinical data indicate a 30-day stroke rate of approximately 1.4% when DLMSs are used in combination with embolic protection, and OCT studies confirm reduced prolapse compared with single-layer stents. Prevention requires an integrated strategy combining lesion-specific characterisation, optimised technique and tailored device selection, while standardised imaging definitions and adequately powered randomised trials with hard clinical endpoints remain research priorities.

## 1. Introduction

### 1.1. Burden of Carotid Artery Stenosis

Carotid artery stenosis is implicated in roughly one-fifth of acute ischemic strokes. Asymptomatic carotid disease is detectable in approximately 7% of women and more than 12% of men older than 70 years, with stenosis above 50–60% generally regarded as the threshold beyond which annual stroke risk increases [1]. Under modern medical therapy, the annual rate of ipsilateral ischaemic events linked to asymptomatic internal carotid artery (ICA) disease has progressively decreased and currently lies in the range of 0.5–1.0% per year. Carotid endarterectomy (CEA) remains the gold-standard treatment for symptomatic disease, reducing the risk of recurrent stroke from above 25% with medical therapy alone to roughly 9–15% in operated patients [2].

### 1.2. The Role of CAS

The role of CAS has been progressively defined by international guidelines. The 2023 European Society for Vascular Surgery (ESVS) guidelines indicate CEA as the preferred revascularization strategy in most symptomatic patients with stenosis ≥ 50% (Class I), while CAS is considered an alternative in patients at high surgical risk, with hostile cervical anatomy, post-radiation stenosis or restenosis after previous CEA, and in centres with documented periprocedural stroke/death rates below 6% for symptomatic and below 3% for asymptomatic patients [2]. Similarly, the 2024 CIRSE Standards of Practice frame protected CAS as the contemporary procedural framework, with systematic use of cerebral protection devices [3]. The most recent ESC clinical consensus statement positions CAS within a multimodal preventive strategy alongside optimal medical therapy and lifestyle modification [4].

The therapeutic equilibrium between revascularisation and intensive medical management has been substantially redefined by the CREST-2 programme (2485 patients with high-grade asymptomatic stenosis ≥ 70%). In the stenting arm, the four-year incidence of perioperative stroke or death plus ipsilateral ischaemic stroke was 2.8% with CAS plus medical therapy versus 6.0% with intensive medical management alone (*p* = 0.02), supporting a clinically relevant additive effect of stenting; in the parallel CEA arm, corresponding incidences were 3.7% and 5.3% (*p* = 0.24) [5]. These findings, alongside earlier randomised evidence in high-surgical-risk populations, have consolidated CAS as a viable alternative when surgery is considered prohibitive.

### 1.3. Plaque Prolapse as a Key Mechanism of Procedural Embolization

In the coronary domain, plaque protrusion or prolapse after stent deployment has been reported in 11–22.5% of cases by intravascular ultrasound (IVUS), with rates further amplified in vessels with positive remodelling, marked eccentricity and large necrotic core [6]. It should be emphasised that these threshold values derive from the coronary circulation and are not directly transferable to the carotid territory, where vessel calibre, plaque composition and flow dynamics differ substantially. In the carotid bed, plaque prolapse acts as a mechanistically distinct, lesion-specific and preventable cause of distal embolization, separable from in-stent thrombus formation, post-procedural hypoperfusion or stent atherothrombosis. Although clinically relevant, prolapse is not synonymous with procedural failure: many through-strut extrusions remain subclinical and translate only into silent diffusion-weighted MRI (DWI-MRI) lesions, while their cumulative load contributes to early neurological complications and to longer-term cognitive consequences [7]. This frames prolapse as a graded, modifiable mechanism of embolization rather than a binary endpoint of procedural success.

### 1.4. Aim and Methods

The aim of the present narrative review is to translate the current understanding of carotid plaque vulnerability and stent–plaque interaction into a practical, lesion-specific framework for prolapse prevention during CAS.

A narrative rather than systematic format was adopted deliberately. Plaque prolapse lacks a standardised definition and is reported across mechanistic, imaging, device-related and clinical studies that differ substantially in design, imaging modality (OCT, IVUS, angiography), stent platform and outcome definitions; this heterogeneity precludes a methodologically sound pooled estimate of a single effect size and instead favours an integrative, practice-oriented synthesis. The review was nonetheless conducted with a structured and reproducible methodology, in keeping with current recommendations for the conduct and reporting of narrative reviews.

Search strategy. Electronic searches were performed in PubMed/MEDLINE, Scopus and the Cochrane Library from January 2000 to December 2025. Controlled vocabulary (MeSH) and free-text terms were combined within and across the following concept blocks: (“carotid artery stenting” OR “carotid stenting” OR CAS) AND (“plaque prolapse” OR “plaque protrusion”) AND (“vulnerable plaque”) AND (“dual-layer stent” OR “micromesh stent”) AND (“optical coherence tomography” OR OCT OR “intravascular ultrasound” OR IVUS), with Boolean operators adapted to the syntax of each database. The search was supplemented by manual screening of the reference lists of included articles, relevant society guidelines and recent systematic reviews.

Study selection and eligibility. Records were screened by title and abstract, and potentially relevant full texts were assessed against the eligibility criteria. Priority was given to prospective registries, comparative cohort studies, randomised data and high-resolution intravascular imaging analyses, with particular attention to studies addressing the plaque–stent interface, stent design and procedural determinants of embolization. Eligible sources comprised randomised controlled trials, prospective and retrospective cohorts, registries, intravascular-imaging and mechanistic studies, together with relevant systematic reviews and meta-analyses; case reports were included only when they illustrated mechanisms not otherwise documented in larger series. Non-English articles and conference abstracts without subsequent full publication were excluded. Selection, data extraction and synthesis were performed by two authors (L.G., L.P.) and verified by a third (E.P.).

Evidence synthesis: Given the absence of comparable quantitative endpoints, the evidence was synthesised narratively and organised thematically around the four domains that structure this review: lesion characterisation, device selection, procedural technique and intra-/post-procedural assessment. Within each domain, priority was given to the most robust and most recent data, and conflicting findings were presented and discussed rather than reconciled statistically. A formal quantitative meta-analysis and a structured risk-of-bias assessment were not undertaken, consistent with the narrative design.

## 2. Definition and Mechanisms of Plaque Prolapse

### 2.1. Definition and Clinical Relevance

Plaque prolapse during CAS is defined as the extrusion of atherosclerotic material through the interstices of the deployed stent into the vessel lumen, occurring at the time of stent expansion or in the post-procedural phase under the effect of pulsatile flow [8]. It is the morphological correlate of a mismatch between the radial expansion of the device and the mechanical resistance of the underlying plaque components: when the lesion contains a soft or fragmented substrate, such as a lipid-rich necrotic core, a thin or ruptured fibrous cap, or intraplaque haemorrhage, the radial force exerted by the stent struts displaces this material outwards, with a fraction forced through the free cell area into the lumen.

Recognition of plaque prolapse depends on the imaging modality used. Conventional angiography may suggest the phenomenon through subtle intraluminal filling defects but its sensitivity is limited. On IVUS, prolapse appears as hyperechoic material projecting between the stent struts, with a protrusion depth > 0.5 mm being the most widely adopted significance threshold, although a unified definition is not yet available [8]. Optical coherence tomography (OCT) provides the highest detail at the stent–plaque interface, with quantitative measurements of protrusion depth, area and longitudinal extent, and discriminates between organised fibrous tissue and the more hyporeflective lipidic or thrombotic material whose embolic potential is greater [9]. The clinical relevance of plaque prolapse rests on its mechanistic link with cerebral embolization: prolapsed material, particularly when of lipidic or thrombotic composition, can fragment under pulsatile flow and embolize, generating periprocedural transient ischaemic attacks (TIAs), ipsilateral stroke or, more frequently, silent DWI-MRI lesions whose cumulative burden has been correlated with cognitive decline [7]. Persistent prolapsed tissue may additionally promote in-stent thrombosis through flow disturbance and contribute to neointimal hyperplasia and late restenosis. Plaque prolapse is therefore a measurable, lesion-specific and device-modifiable mechanism of periprocedural and post-procedural cerebrovascular risk during CAS.

### 2.2. Pathophysiology

Plaque vulnerability emerges from the convergence of intrinsic and extrinsic determinants: the dimensions and consistency of the lipidic core, the tensile strength and collagen architecture of the fibrous cap, and the prevailing inflammatory milieu within the lesion [10]. Once endothelial integrity is compromised, low-density lipoprotein accumulates in the subendothelial space, undergoes oxidative modification and is taken up by macrophage scavenger receptors, giving rise to lipid-laden ‘foam cells’. Progressive apoptosis and secondary necrosis of foam cells and smooth muscle cells contribute to gradual enlargement of the necrotic compartment [10]. Intraplaque haemorrhage (IPH) further destabilises the lesion: extravasation of red cells, leukocytes and platelets fuels a self-sustaining cycle in which erythrocyte breakdown releases iron, cholesterol, glycophorin A and ceroids, driving oxidative stress and inflammation [11].

#### 2.2.1. Thin Fibrous Cap

A fibrous cap thickness below 65 µm on OCT identifies the so-called thin-cap fibroatheroma (TCFA), one of the principal substrates of plaque rupture during CAS. Thin caps typically harbour ~26% macrophage density and low collagen content, with progressive collagen depletion driven by macrophage-derived matrix metalloproteinases [10]. The radial expansion of the stent generates focal mechanical stress that, in such structurally compromised lesions, easily exceeds the tensile capacity of the cap. Cap fracture exposes the underlying necrotic core, which becomes the main source of microemboli [12].

#### 2.2.2. Calcification

Carotid calcific deposits can be classified into three patterns: circumferential, fragmented and calcified nodules. Circumferential calcium can restrict full stent deployment, whereas fragmented calcification and calcified nodules carry the highest risk of plaque prolapse [13]. Where calcium is discontinuous, the radial force of the stent is non-uniform: struts pinch the softer adjacent tissue, and fibro-calcific material may be squeezed between the struts and protrude into the lumen. Calcified nodules represent a distinct entity in which a focal area of nodular calcification disrupts the overlying cap, often with luminal thrombus on top; the mismatch between rigid calcium and soft lipidic components frequently leads to focal cap rupture during stent deployment, releasing both calcific debris and necrotic material into the lumen [14].

### 2.3. Incidence According to Stent Type

**Figure 1:**
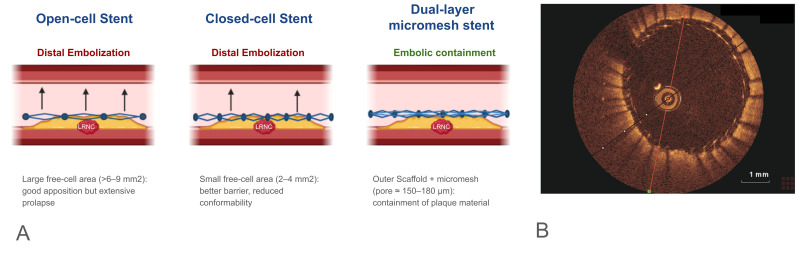
Panel (**A**). Schematic illustration of the relationship between stent cell architecture and plaque prolapse during carotid artery stenting. Open-cell designs are characterised by a large free-cell area that permits extrusion of atherosclerotic material between struts; closed-cell designs provide a denser mechanical scaffold with a smaller free-cell area, at the cost of reduced vessel conformability; dual-layer micromesh designs incorporate a fine inner or outer mesh layer that physically constrains plaque components and limits their displacement into the lumen. Abbreviation: LRNC, lipid-rich necrotic core. The figure is an original schematic and is not drawn to scale. Panel (**B**). Representative intravascular optical coherence tomography (OCT) cross-sectional frames obtained after carotid stent deployment, demonstrating complete circumferential stent apposition to the vessel wall in the absence of detectable plaque prolapse through the stent struts. The high axial resolution of OCT (10–15 µm) enables direct visualisation of the stent–plaque interface and identification of tissue protrusion at a level not achievable by conventional angiography or intravascular ultrasound. Reproduced with permission from [17]. All rights reserved.

The incidence of plaque prolapse during CAS is heavily influenced by stent architecture, with the central trade-off lying between open-cell and closed-cell designs. Open-cell stents have larger free cell areas (typically >6–9 mm^2^), favouring wall apposition and conformability but providing limited mechanical scaffolding; OCT studies show plaque prolapse is detectable in a substantial proportion of cases with this configuration and, in the dedicated OCT series of de Donato et al., was observed more frequently with open-cell than with closed-cell designs (approximately 80% of open-cell versus 53% of closed-cell stents in that 40-patient series, although reported frequencies vary widely with the imaging modality and the prolapse definition applied), with rates further amplified in lesions harbouring a thin fibrous cap or lipid-rich core [8,15]. Closed-cell stents (cell areas 2–4 mm^2^) act more effectively as a physical barrier against extrusion, but their rigid interconnection reduces conformability and, in the tortuous anatomy of the carotid bifurcation, has been associated with higher in-hospital stroke and death [15,16]. It should be noted, however, that the current evidence does not conclusively establish the superiority of either open- or closed-cell free-cell geometry on diffusion-weighted MRI, and the relationship between cell design and cerebral embolization remains a matter of debate [15,16]. The influence of stent architecture on plaque prolapse is illustrated schematically in Figure 1.

## 3. Step 1: Lesion Characterisation: Identifying the High-Risk Plaque

The starting point of any CAS procedure is morphological characterisation of the target lesion to discriminate between a stable and a vulnerable plaque, the latter being more prone to fragmentation or extrusion through the stent struts. This categorisation has direct implications for embolic risk and device selection.

### 3.1. Pre-Procedural Non-Invasive Imaging

Non-invasive evaluation of carotid plaque can be performed with ultrasound (US), computed tomography angiography (CTA) and magnetic resonance imaging (MRI), allowing pre-procedural risk stratification through identification of vulnerability features, a lipid-rich necrotic core (LRNC), IPH, ulceration and intraplaque neovascularization (IPN). The three modalities are presented below in order of decreasing frequency of use in contemporary clinical practice. A two-part consensus document of the European Society of Cardiovascular Radiology (ESCR) has more recently harmonised the acquisition protocols, measurements and structured reporting of carotid CT and MR imaging, providing a standardised basis for plaque characterisation and risk stratification [18,19].

#### 3.1.1. Ultrasound and Contrast-Enhanced Ultrasound

**Figure 2:**
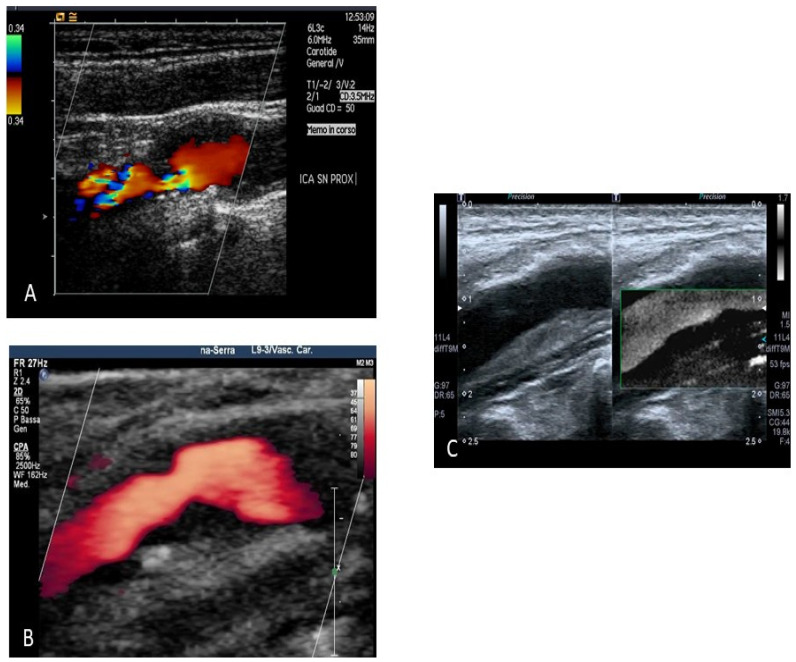
Duplex ultrasound (DUS; (**A**,**B**)) and contrast-enhanced ultrasound (CEUS; (**C**)) of vulnerable carotid plaque. (**A**) Colour Doppler image of the proximal left internal carotid artery showing an eccentric, predominantly hypoechoic (echolucent) bulb plaque with luminal narrowing and a turbulent, aliased flow signal indicating a haemodynamically significant stenosis. Low plaque echogenicity (low grayscale median) is the ultrasonographic correlate of a soft, lipid-rich necrotic core; a juxtaluminal hypoechoic (“black”) area lacking an overlying echogenic cap is, in addition, associated with intraplaque haemorrhage and an increased risk of cap rupture. (**B**) Colour Doppler image demonstrating an irregular luminal contour with a focal recess into which colour flow extends, consistent with plaque ulceration and surface (fibrous-cap) disruption. (**C**) Dual-screen CEUS examination: microbubble contrast sharpens delineation of the luminal surface and can demonstrate intraplaque enhancement; intraplaque neovascularization on CEUS is a recognised marker of plaque inflammation and instability and correlates with intraplaque haemorrhage.

Carotid ultrasonography, with or without contrast enhancement, remains the most frequently used modality in pre-procedural CAS work-up. In B-mode, plaque composition can be inferred through echogenicity analysis, most often expressed by the grayscale median (GSM) score; hypoechoic plaques (lower GSM) reflect higher lipidic content and behave as more vulnerable lesions, with thresholds in the 30–40 range associated with symptomatic disease [20]. Contrast-enhanced ultrasound (CEUS) provides real-time visualisation of intraplaque neovascularization; high-grade neovascularization on CEUS has emerged as an independent predictor of symptomatic stroke, with discriminative performance broadly comparable to high-resolution MRI [21,22]. Practical strengths, including wide availability, low cost, absence of ionising radiation, and ease of repetition, make ultrasound particularly suited to longitudinal follow-up and screening, especially in elderly patients with multiple comorbidities (Figure 2).

#### 3.1.2. Multidetector Computed Tomography Angiography

**Figure 3:**
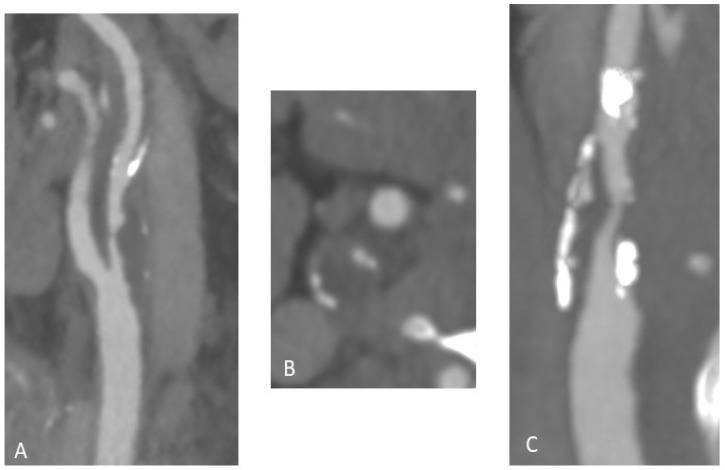
CTA illustrating the spectrum of high-risk carotid plaque morphology that predisposes to peri-procedural distal embolization and post-stent plaque prolapse. (**A**) Curved planar reconstruction (CPR) of the right carotid bifurcation showing a predominantly non-calcified, markedly hypoattenuating plaque at the origin of the internal carotid artery with an irregular luminal contour; the low-attenuation component (mean 30 HU) is in keeping with a lipid-rich/necrotic core, a phenotype classically associated with cap thinning and a high propensity to prolapse through the stent interstices. (**B**) Corresponding axial CTA section through the carotid bulb confirming an eccentric, soft (hypodense) plaque with a thin peripheral rim of calcification and surface irregularity, a finding linked to intraplaque haemorrhage and symptomatic presentation. (**C**) CPR of the right internal carotid artery demonstrating bulky, nodular calcification protruding towards the lumen (calcified-nodule phenotype) with surface irregularity; such heavily calcified, eccentric lesions are relevant to incomplete stent expansion and malapposition.

MDCTA is the second most widely used modality and provides high-quality anatomical images and extensive supra-aortic mapping, with diagnostic performance generally comparable to digital subtraction angiography (sensitivities for ICA stenosis of 70–99%). Plaques can be classified by Hounsfield units into soft, intermediate and calcified types, with histological correlation validating CTA-based identification of fibrous tissue, lipid components and calcium [23]. Although the lipid component can be readily quantified by HU, the visualisation of the fibrous cap and its rupture is less robust than on MRI. Three-dimensional CTA reconstructions may additionally provide information on plaque surface morphology, ulceration and calcium distribution not always evident on standard axial images (Figure 3).

#### 3.1.3. Magnetic Resonance Imaging

Although CTA and contrast-enhanced ultrasound are used more widely in routine clinical practice, dedicated vessel-wall MRI provides the most detailed non-invasive characterisation of carotid plaque composition, with the ability to distinguish individual lesion components and identify features associated with destabilisation, thrombosis and embolization [24]. Sequences such as T1, T2, time-of-flight (TOF), MPRAGE and SNAP enable recognition of LRNC, IPH, fibrous cap disruption and ulceration. A meta-analysis demonstrated that IPH, LRNC and cap thinning/rupture on MRI are each independently associated with substantially elevated hazard ratios for ipsilateral stroke or TIA [25], with subsequent data confirming the same features predict recurrent stroke [20]. Vessel-wall MRI features may further inform device-selection decisions, since markers of plaque vulnerability have been associated with adverse events after stenting [20]. Notwithstanding its diagnostic performance, dedicated vessel-wall MRI remains poorly integrated into routine pre-operative work-up of CAS candidates: limited availability of dedicated coils and protocols, longer scan times, contraindications to magnetic resonance and the need for specifically trained radiologists confine its use largely to high-volume academic centres and selected research settings.

### 3.2. Intraprocedural and Intravascular Imaging

Owing to their superior spatial resolution, IVUS and OCT have emerged as appealing intraprocedural modalities for optimising CAS and limiting embolic risk through more accurate plaque characterisation. Their adoption in the carotid territory remains less widespread than in the coronary district, mainly because of differences in catheter platforms and acquisition protocols.

#### 3.2.1. Intravascular Ultrasound

Modern carotid IVUS systems use rotational or solid-state catheters with profiles between 3.0 F and 3.5 F, advanced over 0.014-inch coronary guidewires through a standard 6-F guide catheter. Image acquisition uses motorised pullback at 0.5–1.0 mm/s during continuous saline flushing, without the need for blood clearance, providing cross-sectional images at 20–60 MHz with axial resolution of 100–200 µm. IVUS recognises IPH, LRNC and a thin fibrous cap, and allows precise quantification of focal stenosis, minimal lumen area, plaque area and plaque burden, all critical for stent selection, expansion and apposition [26]. IVUS is also useful for prolapse prediction: TCFA, defined by plaque burden > 40% and a large necrotic core, identifies a subset of lesions in which tissue extrusion is particularly likely [10].

#### 3.2.2. Optical Coherence Tomography

Carotid OCT uses frequency-domain catheters originally developed for coronary imaging, with a profile of approximately 2.7–2.8 F, compatible with 0.014-inch guidewires through a standard 6-F guide catheter. Unlike IVUS, OCT requires temporary blood clearance to displace red cells; in the carotid bed this is most commonly obtained by non-occlusive flushing with diluted iodinated contrast or carbon dioxide injected through the guide catheter during a rapid (20–40 mm/s) automated pullback [9,27]. The resulting axial resolution of 10–15 µm enables direct measurement of fibrous cap thickness: a value below 65 µm defines TCFA and represents a critical biomarker of embolic risk during stent deployment [10,27]. OCT also visualises IPH, LRNC, macrophage infiltration and calcium with stronger histological correlation than IVUS [8,27]. The two modalities are not interchangeable but complementary: IVUS offers contrast-free, deeper imaging suited to vessel sizing and apposition control, whereas OCT delivers superior resolution at the stent–plaque interface at the cost of additional contrast.

### 3.3. Building a Lesion-Specific Risk Profile

The integration of pre-procedural and intraprocedural imaging findings allows construction of a lesion-specific risk profile that goes beyond simple stenosis grading. A composite stratification logic has been proposed based on the simultaneous evaluation of four parameters: presence of LRNC, evidence of IPH, the status of the fibrous cap (intact, thin or ruptured) and plaque burden quantified through maximum wall thickness (MWT) [20]. Lesions with none or only one feature are classified as low risk; two features identify intermediate risk; three or more features, particularly the combination of IPH, thin/ruptured fibrous cap and large LRNC, define a high-risk lesion with substantially elevated probability of fragmentation, prolapse and periprocedural embolization [20,25]. This stratification has direct implications for device selection: low-risk lesions can be approached with conventional open-cell stent designs and standard distal filter protection, while intermediate- and high-risk profiles support the choice of closed-cell or mesh-covered devices, ideally combined with proximal flow-reversal protection (Table 1).

Table 2 summarises the principal technical differences between IVUS and OCT that are relevant to their intraprocedural use during CAS and to the detection of plaque prolapse.

## 4. Step 2: Device Selection: Matching the Stent to the Lesion

### 4.1. Open-Cell Versus Closed-Cell Stents: Mechanical Rationale and Limitations

The choice between open- and closed-cell carotid stents has historically been the central trade-off in stent selection. Open-cell stents (free cell area > 6–9 mm^2^) provide superior conformability, important in tortuous bifurcation anatomy, but their large pores allow plaque material to extrude through the struts, particularly when soft, lipid-rich or haemorrhagic [15,28]. Closed-cell stents (cell area 2–4 mm^2^) provide a denser scaffold and reduce direct prolapse, but the rigid interconnection of adjacent rings makes them less adaptable to angulated bifurcations and has been associated with higher in-hospital stroke and death in real-world cohorts [15,16]. This intrinsic compromise, containment versus conformability, provided the biomechanical rationale for the development of dual-layer micromesh stent (DLMS) technology [8].

### 4.2. Rationale for the Dual-Layer Micromesh Concept

DLMSs combine a self-expandable nitinol scaffold with a fine inner or outer micromesh designed to act as a physical barrier against plaque protrusion while preserving the conformability of the underlying frame. The mesh, with an effective pore aperture in the range of 150–500 µm depending on the platform, prevents the passage of macroscopic plaque components and contains friable material exposed during deployment, effectively decoupling the scaffolding function (delivered by the open-cell or braided nitinol frame) from the embolic-containment function (delivered by the mesh) [29]. This decoupling represents a substantive shift from the open-cell/closed-cell binary, theoretically allowing simultaneous optimisation of vessel apposition and prolapse prevention in vulnerable lesions [8,29].

### 4.3. Structural Characteristics of Available DLMS Devices

Three DLMS platforms are currently available: CGuard (InspireMD, Miami, FL, USA), Roadsaver/Casper (Terumo, Tyoko, Japan) and the Gore Carotid Stent (W. L. Gore & Associates, Flagstaff, AZ, USA) [29]. They differ in scaffold construction, mesh material and mesh location.

CGuard uses a laser-cut open-cell nitinol frame covered by an external sleeve of polyethylene-terephthalate (PET) MicroNet fibres of approximately 20 µm diameter, producing the smallest effective pore aperture among available DLMS (150–180 µm). The MicroNet expands with the underlying scaffold and adapts to plaque morphology, while the open-cell architecture maintains conformability. Limitations include the modest fluoroscopic visibility of the PET mesh. Because the MicroNet is applied to the abluminal (external) surface of the stent rather than forming an internal lining, it may carry a different—and potentially higher—thrombogenic profile than internally lined designs, an aspect that remains to be fully clarified [29].

Roadsaver/Casper uses two concentric braided nitinol layers, with a finer inner braid (375–500 µm pore aperture) acting as the embolic barrier. The braided architecture confers excellent conformability and outstanding navigability in tortuous anatomies; partial deployment and re-sheathing are possible. The larger pore aperture and the foreshortening that occurs during deployment are operative limitations [29].

Gore Carotid Stent uses a laser-cut open-cell nitinol frame partially covered by an expanded polytetrafluoroethylene (ePTFE) lattice in the mid-body only, with open scaffolding preserved at device ends. This focal containment over the stenotic segment preserves bifurcation flow but limits embolic protection to the central segment.

The clinical implication of these structural differences was directly examined by Umemoto et al. in a prospective OCT comparison: per-frame plaque-prolapse incidence was 10.8% with CGuard versus 20.7% with Roadsaver, consistent with the smaller effective pore aperture of the PET MicroNet [30]. This comparison should be interpreted with caution, as it derives from a non-randomised, single-centre OCT substudy of approximately 42 patients and is therefore subject to selection bias and limited statistical power. Across the dual-layer class the available clinical evidence is device-specific: CGuard has been evaluated in CARENET, PARADIGM, IRONGUARD 2, the Karpenko et al. trial and C-GUARDIANS [31,32,33,34,35]; the Roadsaver/Casper stent in CLEAR-ROAD and ROADSAVER and the analyses of Nerla, Montorsi and Fujii [36,37,38,39,40]; and the Gore stent in the SCAFFOLD trial [41] (Table 3).

### 4.4. Clinical Evidence on Dual-Layer Micromesh Stents

First-generation carotid stents (open- and closed-cell designs) were associated with clinically relevant rates of plaque prolapse on intravascular imaging; in the dedicated OCT study of de Donato et al. (40 patients) prolapse was reported in approximately 80% of open-cell versus 53% of closed-cell stents—a wide range that should not be condensed into a single figure and that varies with the imaging modality and prolapse definition applied [8], a finding that prompted the development of dedicated dual-layer micromesh devices. Three platforms are currently available (CGuard, Roadsaver/Casper, Gore Carotid Stent) and among these.

CGuard has accumulated the largest body of dedicated prospective data, although—as detailed below—comparative meta-analyses indicate broadly similar 30-day outcomes for the Casper/Roadsaver stent and essentially neutral results for the Gore device, arguing against a simple hierarchy among second-generation dual-layer stents. The CARENET and PARADIGM studies established procedural feasibility and safety, including in highly calcific stenoses [31,32]. The IRONGUARD 2 multispecialty registry (733 patients) reported a 30-day stroke rate of 0.54% and 1-year stroke and death rates of 0.68% and 1.22%, respectively [33]. The randomised trial by Karpenko et al. (CGuard vs. Acculink, 100 patients) reduced post-procedural ipsilateral DWI-MRI lesion volume by 78.4% and abolished new ipsilateral lesions, providing the first randomised evidence that a micromesh design can reduce cerebral embolization during CAS [34]; it should be noted, however, that this 100-patient single-centre trial used a surrogate imaging endpoint (diffusion-weighted MRI lesion volume) rather than hard clinical outcomes and does not, in itself, satisfy a formal level-1 (e.g., GRADE) definition. Recent confirmation came from C-GUARDIANS (316 patients, 24 sites), with a 30-day death/stroke/MI rate of 0.95% and any-stroke rate of 0.95% [35].

Roadsaver/Casper has substantial clinical experience: CLEAR-ROAD reported a 30-day major adverse event rate of 2.1% [36]; Nerla et al. confirmed favourable in-hospital and 12-month outcomes in 150 patients [37]; Montorsi et al. showed significantly fewer microembolic signals than the single-layer Carotid Wallstent during the final phases of CAS, particularly with proximal Mo.Ma protection [38]; the large ROADSAVER study (1965 patients) reported a 30-day death/stroke rate of 2.2% [39]; and Fujii et al. confirmed significantly fewer ischaemic complications than the Carotid Wallstent, with no difference in restenosis [40]. The Gore Carotid Stent was evaluated in the SCAFFOLD trial (312 high-surgical-risk patients), with a 30-day death/stroke/MI rate of 3.0%, stroke-or-death rate of 1.5%, and 1-year restenosis of 1.2% [41]; the dataset remains smaller and based on shorter follow-up.

When pooled, DLMS data show a consistent reduction in early embolic burden, with the strongest signal for periprocedural stroke and imaging-defined embolization rather than long-term hard endpoints. An individual-patient meta-analysis (556 procedures) reported a 1-year death/stroke rate of 3.77% and restenosis of 2.1%, more frequent with Roadsaver than CGuard [42]. The systematic review and meta-analysis by Pini et al. (14 studies, 1955 patients) reported a pooled 30-day stroke rate of 1.4% (95% CI 0.9–2.2), with no significant difference between CGuard and Roadsaver/Casper, suggesting a class effect [43]. The CARMEN meta-analysis (68,422 patients, 112 studies) showed a substantially lower 30-day death/stroke/MI rate with second-generation mesh stents than with first-generation devices (1.30% vs. 4.11%; *p* < 0.01); CGuard concurrently improved 12-month ipsilateral stroke and restenosis, Casper/Roadsaver reduced ipsilateral stroke but with a slight increase in restenosis, and the Gore platform produced essentially neutral results [44]. Single-centre contemporary cohorts from Stefanini et al. and Mikelis et al. similarly reported lower 30-day death/stroke/MI and fewer periprocedural neurological complications with double-layer than single-layer stents [45,46]. These findings reinforce the underlying biological rationale, but require cautious interpretation given the predominantly non-randomised, indirect comparisons and substantial heterogeneity in inclusion criteria, protection devices and outcome adjudication.

Direct OCT comparisons further support the device-specific rationale: Umemoto et al. found per-frame plaque-prolapse incidence of 10.8% with CGuard vs. 20.7% with Roadsaver, consistent with the smaller effective pore aperture of the PET MicroNet [30]; indirect support comes from the lower burden of debris recovered in embolic filters with micromesh stents than with single-layer designs [47]. Although the operational definition of prolapse is not fully harmonised, the convergent signal from OCT, embolic filter debris and DWI-MRI surrogates supports its role as a mechanistically meaningful link between plaque vulnerability, stent architecture and periprocedural neurological risk. Current evidence remains limited by the lack of adequately powered randomised trials with hard clinical endpoints, by heterogeneity in the definition and assessment of plaque prolapse, and by selection bias and operator preference that affect real-world comparisons.

### 4.5. Decision Algorithm: Matching the Device to the Lesion

**Figure 4:**
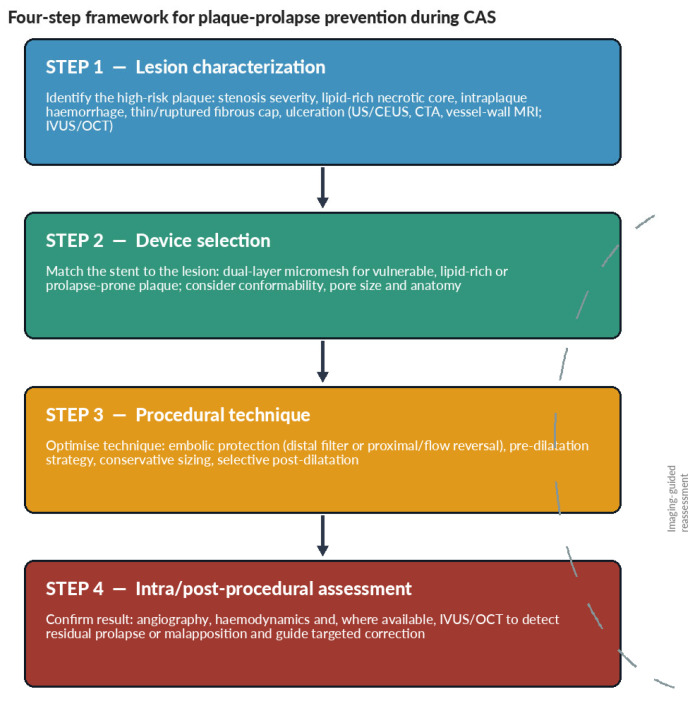
Four-step framework for the prevention and management of plaque prolapse during carotid artery stenting: (1) lesion characterisation, (2) device selection, (3) procedural technique and (4) intra- and post-procedural assessment, with an imaging-guided feedback loop informing device selection. The figure is an original schematic. Abbreviations: CAS, carotid artery stenting; CTA, computed tomography angiography; IVUS, intravascular ultrasound; OCT, optical coherence tomography; TIA, transient ischaemic attack.

Lesion-specific device selection integrates the morphological risk profile defined in Section 3 with the anatomical variables of access and bifurcation angulation. The framework is consistent with current ESVS guidelines [2]. This algorithm is evidence-informed rather than arbitrary, drawing on comparative and registry data on stent design, embolic protection and lesion characterisation [15,16,29,38,44]; its overall structure is summarised schematically in Figure 4.

Short, heavily calcified lesion in a straight segment. The dominant risk is mechanical rupture of the cap over calcified nodules rather than viscous extrusion of a necrotic core; the containment advantage of DLMS is correspondingly less pronounced, and a conventional closed-cell stent or the Gore Carotid Stent may provide adequate scaffolding.

Long, lipid-rich or haemorrhagic lesion with moderate tortuosity. This is the primary indication domain for micromesh technology. Both CGuard and Roadsaver/Casper are appropriate; the presence of IPH on vessel-wall MRI or a thin fibrous cap on OCT argues in favour of the tighter CGuard MicroNet, whereas unfavourable access, severe bifurcation angulation, or a high cervical lesion supports the choice of Roadsaver/Casper [29].

Lesion at an acute bifurcation angle. The braided nitinol construction of Roadsaver/Casper provides better conformability at acute angulation of the ICA origin compared with the CGuard outer frame or with conventional closed-cell stents [29].

Operator experience, institutional device availability and guide-catheter compatibility act as secondary modifiers. Whichever device is selected, the procedural optimisation outlined in Section 5 represents an independent and non-substitutable determinant of outcome.

## 5. Step 3: Procedural Technique Optimisation

### 5.1. Vascular Access

Vascular access is increasingly recognised as integral to CAS optimisation. Transfemoral access remains widely used, although several high-volume centres now adopt transradial access as a first-line strategy in selected subgroups, particularly when hostile arch anatomy, marked supra-aortic tortuosity, a bovine arch variant or extensive aortic atheroma are anticipated. Available series and meta-analytic data suggest that transradial access is feasible and safe compared with transfemoral, although crossover may be more frequent and catheter support less robust in complex anatomies; a systematic review reported no significant differences in stroke, death, MI, TIA or access-site complications [48]. Transcarotid access with flow reversal can substantially limit arch manipulation in patients with high embolic risk or unfavourable arch morphology. Aortic arch anatomy strongly influences carotid stent deliverability, particularly in type II–III arches. In hostile arches, an adequate guiding system, including supportive guide catheters or long sheaths, is essential to provide proximal stability and limit repeated passes across emboligenic segments.

### 5.2. Pre-Dilatation

Pre-dilatation is one of the most debated steps, especially when the goal is minimising plaque prolapse and distal embolization. When judged necessary, balloons < 5 mm in diameter are generally recommended to limit stroke or TIA risk [2]. Inflation before stent deployment can rupture the fibrous cap, disrupt the plaque surface and release embolic debris during a phase in which the lesion is not yet sealed. Aggressive pre-dilatation is therefore generally discouraged, particularly in lipid-rich or otherwise vulnerable plaques [10].

### 5.3. Lesion Crossing

During crossing, the use of roadmap or image-fusion overlay techniques is reasonable; the lesion can then be gently traversed with a 0.014-inch soft-tip straight crossing wire shaped according to anatomy and plaque characteristics.

### 5.4. Embolic Protection Devices

Embolic protection devices (EPDs) retain a central role. Distal filters, proximal occlusion systems and flow-reversal strategies all aim to reduce cerebral embolization during lesion crossing, ballooning, stent deployment and post-dilatation. The 2023 ESVS guidelines indicate that cerebral protection should be considered during CAS [2] and the 2024 CIRSE Standards include protected CAS as the contemporary procedural framework [3]. Whether DLMS can ultimately reduce the need for EPDs is debated, but the available evidence does not support abandoning embolic protection. Indeed, the dual-layer registries that report low embolic event rates—including IRONGUARD 2, ROADSAVER and CARENET—mandated the use of an embolic protection device, so that the pooled 30-day stroke rate of approximately 1.4% reflects the combination of a dual-layer stent and embolic protection rather than the stent alone. In a network meta-analysis, proximal balloon occlusion outperformed distal filters in reducing angiographic evidence of embolization, although no significant difference in clinical stroke or TIA was demonstrated between protection strategies [49]; the combination of a dual-layer stent with proximal flow reversal has been associated with fewer microembolic signals than a single-layer stent [38]. EPD selection should be individualised based on lesion morphology, vessel anatomy and operator experience. Proximal protection (e.g., Mo.Ma) may be particularly attractive in high-risk plaques, since protection is established before lesion crossing, although proximal occlusion can be poorly tolerated and is contraindicated in advanced common carotid disease, significant external carotid disease or contralateral occlusion with inadequate collateralization.

### 5.5. Stent Sizing and Selection

In standard practice, stent diameter is selected 1–2 mm larger than the reference vessel diameter. In the carotid territory, however, sizing should not be approached as a purely geometric exercise: oversizing may compress the lipid core and fracture friable components against the scaffold, potentially favouring extrusion through the struts, while undersizing may result in incomplete apposition. With DLMS, a more conservative sizing strategy is often reasonable, usually favouring a diameter only slightly above the reference artery, since these devices provide enhanced plaque coverage by design. The principle of conservative, coverage-oriented sizing—prioritising adequate plaque coverage over maximal luminal gain—is consistent with current procedural guidance [2]. CGuard appears highly flexible and well suited to tortuous anatomies, even if its navigability before deployment may be limited; Roadsaver, by contrast, may show lower conformability to the carotid bifurcation, with a possible impact on post-implant geometry whose clinical implications remain to be established, and offers good navigability during delivery, with a degree of foreshortening upon deployment. It should be acknowledged that platform differences mean that “dual-layer” is not a uniform category: decoupling radial scaffolding from embolic containment is conceptually attractive but is not realised uniformly across devices, and describing this class as the most advanced device-based option reflects a class-level rationale and a pooled embolic-protection signal rather than demonstrated superiority of every device over closed-cell stents in every clinical setting.

### 5.6. Post-Dilatation

Post-dilatation is commonly performed to optimise stent expansion and apposition but carries a clinically meaningful trade-off in embolic risk and plaque protrusion. Several studies show that its impact on outcomes is mediated by two additive factors, increased embolic showering and haemodynamic depression. Post-dilatation may also mechanically squeeze the plaque through the scaffold, increasing prolapse. Obeid et al., in 3772 patients, showed that combined pre- and post-stent ballooning was associated with a 2.1-fold increase in haemodynamic depression and a 2.4-fold increase in perioperative stroke and death compared with pre-stent ballooning alone [50]. Post-dilatation should therefore be performed selectively, generally only when completion angiography reveals residual stenosis above 30%, since the procedural goal in CAS is adequate plaque coverage rather than maximal luminal expansion [2]. According to OCT-based evidence, post-dilatation in unstable plaques may improve luminal gain and reduce large protrusion, but at the cost of increased small tissue prolapse, likely reflecting plaque fragmentation [51]. These observations can be reconciled as follows: routine high-pressure post-dilatation is discouraged, whereas a single, targeted, low-pressure inflation reserved for focal malapposition or a residual stenosis greater than 30% on completion angiography or intravascular imaging represents a distinct and selective manoeuvre. The default should therefore be to avoid post-dilatation and to reserve it for documented residual stenosis or focal malapposition, ideally confirmed by IVUS or OCT, using a slightly undersized balloon at low pressure (Table 4).

## 6. Step 4: Intraprocedural and Post-Procedural Assessment

### 6.1. Intraprocedural Optical Coherence Tomography

OCT provides axial resolution of 10–15 µm at the stent–plaque interface, allowing direct quantification of protrusion parameters invisible to angiography. In the carotid bed, OCT acquisition requires temporary blood clearance, most commonly obtained by injection of diluted iodinated contrast or carbon dioxide through a standard 6-F guide catheter; no dedicated equipment beyond that used for coronary OCT is required [9]. The principal OCT-derived parameters describing the stent–plaque interface after CAS were established by de Donato et al. and validated in dedicated carotid series [8]: maximum protrusion depth, cross-sectional protrusion area on the worst-affected frame, and morphological appearance of protruding material (organised hyperreflective fibrous vs. unorganised hyporeflective lipidic, the latter associated with greater embolic potential). The most widely applied threshold for clinically significant prolapse is a maximum depth ≥ 0.5 mm on at least one longitudinal reconstruction frame, although consensus standardisation remains an unmet need (Section 7). Routine intraprocedural carotid OCT is not yet standard of care, primarily because of additional contrast burden and the operator learning curve; its use is reasonably reserved to high-volume centres for morphologically high-risk lesions and to the prospective registry or trial setting [53].

### 6.2. Intraprocedural Intravascular Ultrasound

IVUS offers two practical advantages over OCT in the carotid setting: it does not require blood clearance, and no additional iodinated contrast is needed. Axial resolution is, however, coarser than OCT (150–200 µm), which limits detection of shallow protrusions and precludes direct measurement of fibrous cap thickness. The dominant intraprocedural role of IVUS in dual-layer stenting is the guidance of post-dilatation optimisation: when IVUS identifies persistent malapposition or asymmetric expansion, targeted low-pressure balloon inflation can be performed under real-time monitoring to confirm correction without inducing additional plaque extrusion through residual stent interstices. Squizzato et al. provided indirect supporting evidence showing that embolic filter debris was significantly lower with micromesh stents than with open-cell or closed-cell designs in 481 procedures [47].

### 6.3. Criteria for the Optimal Post-CAS Result

An optimal post-procedural result is defined by angiographic, haemodynamic and, where intravascular imaging is available, morphological criteria. Angiographically, residual luminal narrowing below 30% by NASCET criteria in the absence of significant filling defects is acceptable, but angiography has a low sensitivity for prolapse and stent malapposition [2,8]. When intravascular imaging is performed, additional criteria apply: protrusion depth below 0.5 mm; complete circumferential apposition; absence of significant edge dissection; and symmetric stent expansion. Focal incomplete apposition, particularly at the ICA origin, predisposes to flow disturbance and potential in-stent thrombus, justifying targeted low-pressure balloon inflation if identified intraprocedurally, balanced against the risk of additional plaque displacement.

### 6.4. Clinical Consequences of Unrecognised Plaque Prolapse

Plaque prolapse not identified by conventional angiography may produce a spectrum of adverse consequences extending from the immediate periprocedural period into medium-term follow-up. The most immediate is periprocedural TIA or ipsilateral stroke, resulting from direct embolization of extruded material or from thrombus forming on exposed plaque [54]. New DWI-MRI lesions, a sensitive surrogate of periprocedural embolic load, have been reported in 30–70% of patients after CAS with conventional single-layer stents [55]. Although most are asymptomatic at detection, the cumulative silent ischaemic burden has been correlated with cognitive decline on serial neuropsychological testing, an observation of growing relevance given expanded indications for CAS in asymptomatic patients [7]. Beyond the periprocedural window, persistent prolapsed tissue may theoretically contribute to neointimal hyperplasia and to flow disturbance, although direct evidence linking prolapse to long-term restenosis or in-stent thrombosis under contemporary DAPT regimens remains limited.

## 7. Limitations and Future Perspectives

### 7.1. Limitations of the Current Evidence

Despite the biological coherence of the prolapse-prevention framework, the evidence base retains structural weaknesses. Only one adequately conducted randomised controlled trial has directly compared a DLMS (CGuard, InspireMD, Miami, FL, USA) against a conventional single-layer device (Acculink); its primary endpoint, however, was a DWI-MRI surrogate rather than a hard clinical outcome, and enrolment was restricted to 100 patients at a single high-volume centre, substantially limiting external validity [34,44]. The recently published CREST-2 trials confirmed the benefit of CAS over intensive medical therapy alone in asymptomatic severe stenosis, but were not designed to address device-specific questions, and the vast majority of interventions used first-generation platforms [5]. A second structural limitation concerns the absence of universally accepted thresholds for significant plaque prolapse on intravascular imaging, with OCT protrusion-depth cut-offs ranging from 0.2 to 0.5 mm and heterogeneous IVUS criteria coexisting in the literature [8]. Finally, large-scale meta-analytic data have documented clinically relevant outcome differences among individual dual-layer platforms rather than a uniform class effect, a heterogeneity that current non-randomised comparisons cannot adequately resolve [44].

### 7.2. Future Perspectives

Several converging developments are likely to refine the prolapse-prevention paradigm. Standardised reporting systems for carotid plaque imaging, supported by recent expert consensus efforts, may help harmonise pre-procedural risk assessment across imaging modalities, with incremental prognostic value beyond stenosis quantification [20]. Machine-learning methods for automated segmentation and compositional analysis of carotid plaques from CTA and vessel-wall MRI have progressed from proof-of-concept to multicentre validation. The only randomised micromesh trial reported to date is the single-centre study of Karpenko et al., which was powered on a surrogate imaging endpoint [34]; adequately powered, multicentre randomised trials comparing dual-layer stents against conventional platforms on hard clinical endpoints are still awaited and represent a key research priority. On the device side, next-generation bioresorbable scaffolds are under active evaluation in peripheral arteries and might extend to the carotid territory; complementary directions include polymer coatings to accelerate endothelialisation and mesh architectures with variable pore geometry matched to local plaque composition. Priority research needs include adequately powered randomised trials with hard clinical endpoints, central core-laboratory adjudication using harmonised prolapse definitions, and prospective integration of standardised pre-procedural plaque classification systems into device-selection algorithms.

## 8. Conclusions

Plaque prolapse represents a mechanistically defined and device-modifiable pathway linking atherosclerotic plaque vulnerability to periprocedural cerebral embolization during CAS. Its prevention is best conceptualised as a sequential four-step framework, with each phase acting as a non-substitutable determinant of outcome: lesion-specific morphological characterisation through appropriate non-invasive and, where feasible, intravascular imaging; rational device selection based on the interaction between plaque composition, anatomy and stent geometry; conservative procedural technique in pre-dilatation, sizing and post-dilatation; and targeted intra- and post-procedural assessment.

Within this framework, DLMS currently constitute the most advanced device-based solution for the containment of vulnerable plaque material, with consistent evidence of reduced periprocedural embolic load translating into low 30-day stroke rates across registries and pooled analyses. The three principal platforms are not interchangeable, however, and the magnitude of benefit depends on appropriate device-to-lesion matching rather than a generic class effect [29]. Procedural technique retains independent prognostic weight: no stent architecture, however refined, can compensate for oversized deployment, aggressive post-dilatation or inadequate embolic protection. The field now requires a coordinated evidentiary effort: adequately powered randomised trials with hard clinical endpoints, harmonised intravascular imaging definitions of plaque prolapse, and prospective incorporation of pre-procedural plaque classification systems into device-selection algorithms. Until such evidence becomes available, lesion-specific individualisation of CAS technique, anchored in the dual-layer micromesh design paradigm, represents the most robust current strategy to limit plaque prolapse and its associated embolic consequences.

## Figures and Tables

**Table 1:** Imaging modalities for the pre-procedural and intraprocedural characterisation of vulnerable carotid plaque features relevant to CAS. For each feature, the gold-standard technique is indicated first, with alternative modalities and the conditions under which they should be preferred.

Plaque Feature	Clinical Significance	Best Detected by (Pre-Procedural)	Best Detected by (Intraprocedural)
Intraplaque haemorrhage IPH	Strongest independent predictor of future ipsilateral stroke or TIA (HR 4.59, 95% CI 2.91–7.24) [25].	MRI (gold standard); MPRAGE/TOF identify IPH as hyperintense signal [24,25] CEUS when MRI unavailable [21].	IVUS (gold standard); HD-IVUS identifies IPH in cross-section. OCT when IVUS unavailable; higher resolution but requires flush [8,9].
Lipid-rich necrotic core LRNC	Substrate for cap rupture and source of embolic material; independently associated with ipsilateral stroke [25].	MRI (gold standard) with high contrast-to-noise. CTA when MRI unavailable; HU thresholds correlate with histology [23].	OCT (gold standard); hyporeflective region with diffuse borders. IVUS when OCT unavailable [8,9].
Thin/ruptured fibrous cap	Defines TCFA (cap < 65 µm); precursor of acute embolic events [10].	MRI for cap thinning/disruption [24]. CEUS when MRI unavailable [21].	OCT (gold standard); only modality directly measuring cap thickness < 65 µm [27].
Calcification (pattern matters)	Calcified nodules and fragmented calcium increase prolapse risk; circumferential calcium impairs expansion [13,14].	CTA (gold standard) for calcium pattern classification [23].	IVUS (gold standard) for in-procedure calcium burden. OCT complementary; tends to underestimate calcified areas [8].
Plaque burden/max wall thickness MWT	Composite indicator of plaque mass; large burden associated with vulnerability and prolapse.	MRI for vessel-wall quantification [20]. US/CTA for routine assessment.	IVUS (gold standard) for cross-sectional plaque burden [26].
Intraplaque neovascularization IPN	Marker of inflammation, IPH and instability.	CEUS (gold standard); high-grade neovascularization predicts symptomatic events [21,22].	Not routinely assessable.
Ulceration	Indicator of cap disruption and prior thrombo-embolic activity.	CTA (gold standard) with multiplanar reformat [23]. MRI complementary [24].	OCT for surface morphology when intravascular imaging used [9].

Acronyms: CAS, carotid artery stenting; CEUS, contrast-enhanced ultrasound; CTA, computed tomography angiography; HD-IVUS, high-definition intravascular ultrasound; HR, hazard ratio; HU, Hounsfield units; IPH, intraplaque haemorrhage; IPN, intraplaque neovascularization; IVUS, intravascular ultrasound; LRNC, lipid-rich necrotic core; MPRAGE, magnetisation-prepared rapid acquisition gradient echo; MRI, magnetic resonance imaging; MWT, maximum wall thickness; OCT, optical coherence tomography; TCFA, thin-cap fibroatheroma; TIA, transient ischaemic attack; TOF, time-of-flight; US, ultrasound.

**Table 2:** Comparison of intravascular ultrasound (IVUS) and optical coherence tomography (OCT) for intraprocedural imaging during carotid artery stenting.

Feature	IVUS	OCT
Axial resolution	100–150 µm	10–20 µm
Tissue penetration	4–8 mm	1–2 mm
Imaging source	Ultrasound (rotational or solid-state, ~20–60 MHz)	Near-infrared light (frequency-domain)
Blood clearance	Not required	Required (saline/contrast flush)
Contrast medium	None	Iodinated contrast for luminal flush
Catheter profile	~3.0–3.5 F	~2.7–2.8 F
Main strengths	Deep penetration; images the full plaque and outer vessel wall; no blood clearance needed	Very high resolution; superior detection of thrombus, thin-cap fibroatheroma, malapposition and plaque prolapse
Main limitations	Lower spatial resolution; limited surface and cap detail	Shallow penetration; cannot image the full depth of large plaques; requires blood clearance
Principal role in CAS	Vessel sizing, stent apposition and assessment of gross prolapse	Detailed detection of in-stent plaque prolapse, malapposition and tissue protrusion

CAS, carotid artery stenting; F, French gauge; IVUS, intravascular ultrasound; OCT, optical coherence tomography. Values are indicative ranges drawn from contemporary intravascular-imaging literature and may vary with the specific catheter and system used.

**Table 3:** Structural and design characteristics of the three commercially available dual-layer micromesh carotid stents.

Feature	CGuard (InspireMD)	Roadsaver/Casper (Terumo)	Gore Carotid Stent (W. L. Gore)
Structure	Nitinol frame + PET MicroNet sleeve	Two concentric nitinol braided layers	Nitinol frame + partial ePTFE membrane
Frame architecture	Laser-cut, open-cell	Braided, closed-cell behaviour	Laser-cut, open-cell
Mesh material	PET fibre (20 µm diameter)	Braided nitinol (inner layer)	ePTFE lattice (mid-body only)
Mesh position	External (sleeve over frame)	Internal (inner braid)	External, partial coverage
Nominal pore aperture	150–180 µm	375–500 µm	≈500 µm
Effective inner cell area	≈0.02–0.04 mm^2^	≈0.2–0.3 mm^2^	≈0.25 mm^2^ (membrane zone)
Delivery profile	6 F	5 F; partial deployment and re-sheathing possible	6 F
Available sizes	6–10 mm × 20–40 mm	5–10 mm × 16–40 mm (22–47 mm with flares)	6–10 mm × 20–40 mm
Key advantages	Tightest pore coverage; flexible mesh adaptation; preserved frame conformability	Low-profile delivery; outstanding conformability in tortuous anatomy	Focal plaque isolation over the stenotic segment; open scaffolding at device ends
Key limitations	Limited radiopacity of the PET mesh	Larger pore aperture; foreshortening during deployment	Containment limited to the mid-body; smaller dataset
Key clinical evidence	CARENET, PARADIGM, IRONGUARD 2, Karpenko et al. trial, C-GUARDIANS [31,32,33,34,35]	CLEAR-ROAD, ROADSAVER; Nerla, Montorsi, Fujii [36,37,38,39,40]	SCAFFOLD [41]

PET, polyethylene terephthalate; ePTFE, expanded polytetrafluoroethylene. Pore aperture, fibre and cell-area values are derived from manufacturer and bench-test data [29].

**Table 4:** Pragmatic instructions for use (IFU) for plaque prolapse prevention during carotid artery stenting, organised by procedural phase. Recommendations summarise consensus from current ESVS and CIRSE practice statements together with operator experience; red flags identify settings in which DLMS deployment may be inappropriate or in which heightened operator vigilance is warranted.

Phase	Recommendation	Red Flag
Pre-operative	• Careful patient selection: morphological characterisation of the plaque (US/CTA first-line, MRI vessel-wall and CEUS where available); • DLMS preferred for vulnerable lesions (lipid-rich, IPH, thin cap); CGuard for greater conformability needs; • Roadsaver/Casper when navigability and a low-profile delivery system are priorities.	• CAS during the acute stroke phase: this calls for caution and careful case selection rather than representing an absolute contraindication to DLMS, given the dedicated multicentre experience with a MicroNet-covered stent reported in SAFEGUARD-STROKE [52]; • Severe contralateral occlusion with inadequate collateralisation when proximal protection is planned.
Intra-operative	• Cross the lesion gently using roadmap or image-fusion overlay, with a 0.014-inch soft-tip wire; • Avoid pre-dilatation when possible; if mandatory, use a balloon ≤ 4 mm with slow inflation/deflation at low pressure (≈4 atm) and 1:1 sizing; • Stent sizing slightly above the reference vessel diameter (no aggressive over-sizing). • Avoid systematic post-dilatation; reserve it for residual stenosis >30%; • Embolic protection always recommended; consider proximal flow reversal (Mo.Ma) in high-risk plaques.	• High-grade stenosis crossed too easily on the wire (suggests very soft or unstable substrate); • Low calcium burden combined with hypoechoic/lipid-rich morphology; • Persistent malapposition or asymmetric expansion on intravascular imaging despite optimised deployment.
Post-operative	• Standard DAPT according to current guidelines; • Optimal DAPT duration after DLMS still uncertain, individualised; • Early clinical and imaging surveillance for residual stenosis, plaque protrusion or in-stent thrombosis.	• Persistent neurological symptoms within the first 24–48 h: consider DWI-MRI and Duplex re-evaluation. • New residual stenosis or filling defect on follow-up imaging.

## Data Availability

The present study is a narrative review based on previously published literature. No new datasets were generated or analysed; therefore, data sharing is not applicable.

## Abbreviations

The following abbreviations are used in this manuscript:

CAS	Carotid artery stenting
CEA	Carotid endarterectomy
CEUS	Contrast-enhanced ultrasound
CIRSE	Cardiovascular and Interventional Radiological Society of Europe
CTA	Computed tomography angiography
DLMS	Dual-layer micromesh stent
DWI-MRI	Diffusion-weighted magnetic resonance imaging
EPD	Embolic protection device
ePTFE	Expanded polytetrafluoroethylene
ESVS	European Society for Vascular Surgery
GSM	Grayscale median
HU	Hounsfield units
ICA	Internal carotid artery
IPH	Intraplaque haemorrhage
IPN	Intraplaque neovascularization
IVUS	Intravascular ultrasound
LRNC	Lipid-rich necrotic core
MDCTA	Multidetector computed tomography angiography
MRI	Magnetic resonance imaging
MWT	Maximum wall thickness
OCT	Optical coherence tomography
PET	Polyethylene terephthalate
TCFA	Thin-cap fibroatheroma
TIA	Transient ischaemic attack
TOF	Time-of-flight
